# Recherches Expérimentales sur le Développement de la Graisse Pendant I'Alimentation des Animaux

**Published:** 1847-01

**Authors:** 


### ART. XI.

1. *Recherches Expérimentales sur le Développement de la Graisse pendant l'Alimentation des Animaux.* Par M. BOUSSINGAULT. (Annales de Chimie, tom. xiv, 1845.)
2. *Der Chemische Process der Respiration.* Von JUSTUS VON LIEBIG. (Annalen der Chemie, Band lviii, 1846.)
3. *Experimental Researches on the Food of Animals and Fattening of Cattle, with Remarks on the Food of Man.* By ROBERT DUNDAS THOMSON, M.D.—1846. 8vo.

THERE are no terms which have suffered more abuse in the hands of those who are imperfectly educated in the science of observation than those of *practice* and *experience.* An example illustrative of this affirmation will place it in a clearer light than any abstract reasoning could accomplish. It has been found by many practical and experienced men, that a very large proportion of patients treated by them while labouring under Indian cholera has terminated fatally, while to another class, equally entitled to the same appellation, the issue of their experience has been diametrically opposite. Instead of disaster they have met with recovery, and have without delay announced their success as the result of their superior method of treatment. Now here we have presented to our attention two common illustrations of the results of practical experience, each of which is incompatible with the other. If no other considerations are taken into account than the simple facts that such has been the experience of two practitioners, the unfortunate position, the *opprobrium medicorum,* the impossibility of arriving at a decision among disagreeing doctors is attained. We believe we do not exaggerate, when we affirm that this mode of drawing conclusions respecting the efficacy of medical treatment, is a very common method of procedure, and that a large proportion of the cures which are daily recorded in respect to the treatment of fevers, and other diseases characterized by a distinct type, are not, in reality, errors of fact, but simple statements of the cases which have occurred, and of their termination, without reference to any important element which can enable us to decide as to the natural law of the disease, and, by consequence, as to the possible influence of medicaments administered to remove any natural symptom or excess of natural action. By this mode of viewing the question, then it is at once obvious that we admit the possible, nay, probable accuracy of two practitioners treating the same disease; the one with the most fatal results, the other with the most successful issue. But we do not concede that by practical experience or common sense alone, in the ordinary use of these terms, these two incongruities can be reconciled. Mere common sense, or the general opinion of mankind, although absolutely requisite to the philosopher, is, as Sir John Leslie has well observed, "a very suspicious standard of appeal in matters of science." We believe medicine, if followed by a philosophical course, to be one of the noblest of the sciences, and therefore subject, as much as any other, to suffer by a bare appeal to so limited a source of judgment. The philosopher in other sciences would not be contented with a mere determination of the facts, as in the case cited, and an inference; but he would investigate all the connecting circumstances, and eliminate every element which would appear to lead to an erroneous conclusion. He would thus endeavour to ascertain what are the usual phases of the disease, and in what respect they differ from those which it is concluded have been subjugated by medical appliances. He would thus be under the necessity of making an extensive series of observations, that is, in medicine, of collecting a large number of cases, carefully bearing upon certain distinguishing features of the disease. In the example of cholera, which we have cited, he would find that out of 100 persons affected with this disease, one fourth die on the first day of infection, or 25·219, while only 0·3 recover; on the second day 21·7 die and 1·25 recover; on the third day 10·5 die and 4·23 recover; on the fourth day 6·3 die and 4·9 recover; while on the nineteenth day only 1 dies and 12·5 recover; and after the 25th day no death occurs, and all who are still sick recover. (Farr, Medical Annual, 1839.) The anomalous and contradictory position in which the two practitioners stood, who were previously noticed, after the knowledge of this law, is now explicable. It would now be quite legitimate to infer, as the data were not supplied by the practitioners themselves, that the cases which all perished, were those of persons newly affected, while those which recovered, with few or no exceptions, were old cases, which had survived the dangers of the first stages of the disease. This example is sufficient to impress upon us the importance of an acquaintance with the laws of the animal economy, not only in health, but also in disease, before we can consider ourselves competent to decide upon the amount of influence which we can exercise over disease, or judge of its weak and impregnable points under the administration of curative means.

The same mode of reasoning is applicable to the means requisite for preserving the systems of animals in a healthy condition. Mere experience, after the fashion in which that expression is commonly employed, has utterly failed to enable us to lay down strict rules for the regulation of diet. The glimpses of truth which, in this respect, peer out in the distance, have been derived from other than men of mere practical experience. The foundation-stone of a true theory of diet was laid by a religious father (Beccaria), and the more modern advocate of a philosophical view of nutrition was a physician (Dr. Prout), who rose to fame and improved our knowledge of the healing art by a thorough acquaintance and masterly application of the *principles* of chemical science. Mere experience declares that many practices are perfectly wholesome which it is difficult to find any other arguments to support. The use of alcohol among human beings, in moderation, as it is termed, is one of these, and even in towns it has become customary to stimulate the systems of cattle by means of pot ale, under the idea of increasing the amount of milk. Now, if this pot ale have any stimulating influence, it must derive such power from the presence of alcohol, as the fluid contains no casein, or, if so, but an insignificant portion, and hence is not endowed with any nutritive energy. (Experimental Researches, p. 12.) There cannot be a doubt that in general we are far more merciful in the treatment of the brute than of the rational creation. What would be the public expression, if the owner of a horse were to sentence his dumb assistant to receive five dozen lashes with the apparatus usually employed to castigate the rational soldier, and carry the sentence of his court-brutal into execution in a locality to which the people had access? or what would be the indignant criticisms of the press? and would not the very dregs of the population rush to the rescue, if houses should be licensed for the purpose of legally disposing of fermented fluids for the consumption of the inferior animals? Would one solitary being exist who should advocate such a palpable specimen of criminality? Yet, is it not obvious to the least experienced, that the brute creation possesses more physical endurance, and more power to resist offensive reaction, than the strongest of the intellectual class of beings? What then has been the result of the use of such stimulating fluids when administered for any considerable period to cattle? Mr. Harley, who kept the celebrated dairy at Glasgow, and has published an interesting description of his establishment, informs us that as the season advanced he was in the habit of feeding his cows with grains and distiller's wash, in addition to a certain portion of succulent food; "but they were apt to make the cattle grain sick, as it is termed, and to prove injurious to the stomach of the animal. It has been ascertained that if cows are fed upon grains or distiller's wash, their constitution will quickly be destroyed. Cattle thus fed should not be kept longer than eight or ten months." We believe this opinion of a man of great experience in the feeding of cattle to be borne out by that of others who have acquired knowledge in a similar practical school. If such, then, is the result of the administration of moderate doses of fermented fluids to the cow, an animal whose physical constitution is proverbially of no peculiar delicacy, it is natural to ask if it be not equally or at least somewhat noxious to the weaker physical condition of man. It is no argument in favour of any such experiment upon human life that existence does not terminate on its adoption, or that the symptoms of some frightful disease are not instantly ushered in; the seeds of future mischief may be slowly and gradually sown by even one or succeeding experiments, and may only lie dormant until other repetitions shall cause them to spring forth into living activity. (Exp. Res., p. 7.) Our argument, then, is that it is impossible in the present state of our knowledge to declare that even moderate quantities of certain substances, usually admitted as wholesome articles of diet, do not tend to impair the integrity of the human organism. We have seen, in reference to cattle, that a fluid considered healthy for the system of man, is unequivocally pronounced to shorten their earthly span. Is it probable then, we should ask, that to man the same diet should be innoxious, and that no elements of future mischief have been thereby constituted. At present we are unacquainted with the secondary results of the application of fermented fluids to the interior of the system. We know that it stimulates the nervous system, and that its influence decays with tolerable rapidity; but with what chemical decompositions this subsidence is accompanied, has not yet been ascertained; and hence we are not in a condition to predicate whether the products of the secondary changes in alcohol may not be calculated to produce even a worse influence on the system than that which is effected on the nervous system by the primary stimulating and the resulting depressive action of this powerful agent.

There is a speculation with regard to the possible formation of alcohol from the sugar of the food in the vascular system, the investigation of which would lead to much important exposition of fact. Mitscherlich has given it as his opinion, that the sugar swallowed by animals may be converted into alcohol in the intestines or vessels into which the absorbed food passes. The possibility of this change, it is true, cannot be denied, but there is one objection to the probability of the alcohol remaining for any length of period in this form in the body, that the temperature of an animal would immediately tend to convert it into acetic acid. There is, however, a curious fact which we have met with, and which would appear to corroborate the suspicion of Mitscherlich; it is a circumstance related in a note by the late Dr. Oudney, in his African travels: "Several of our camels are drunk to-day; their eyes are heavy and want animation, gait staggering, and every now and then falling as a man in a state of intoxication. It arose from eating dates after drinking water; these probably pass into the spirituous fermentation in the stomach."

Mere experience, in the ordinary sense of the expression, is not calculated any more than in the case which we have previously cited to inform us as to the wholesomeness or noxiousness of the practice of consuming food in a state of partial decomposition. On the contrary, if we can judge by the extent to which the use of decayed cheese and putrid game is employed, we should be inclined to infer that experience is decidedly in favour of their consumption. It is necessary before we can pass a faithful judgment upon a question so deeply based as this, to take an extensive view of the constitution, of the animal system, and of the mode in which it is preserved in all its integrity from waste and decay. One of the strongest proofs of the inability of common experience to give a valuable opinion upon such a point as this, is the remarkable fact that we do not remember to have seen in any work of original merit upon dietetics the question discussed as to the propriety of permitting or withholding from human beings food, such as we have described to be in a rapid state of decomposition. Yet it is well known that cases of death have frequently occurred from the use of decayed cheese. The deteriorating action in these cases was produced by the influence of a poison, which communicated its baneful state of decomposition to the human system. The body becomes dry and emaciated, and death closes the scene, leaving a mummy-like wreck behind. In such an example, decaying cheese is discovered to be unwholesome, because its action has been allowed to proceed to excess; in short, because a telling experiment, to use a familiar phrase, has been performed. But to argue that it is only when such striking impressions are made upon the senses that we are to suspect disease, would be as fallacious as the conduct of the philosopher who should insist that such phenomena as the showy combustion of gases exhibited on the lecture-table, or in the laboratory of the chemist, exercised a more important influence in the domain of science than the long-continued, secret, and almost insensible actions of a weak battery, which are known to be analogous to the most important electrical operations of nature; far more important indeed, and much more extensive in their consequences, than the most awe-striking lightning or the most powerful reverberations of a thunderstorm. It is, therefore, we conclude with some show of reason, that the man of science advises the physician to direct his attention to principles, as well as to ocular demonstration, guided by mere common sense. It becomes the duty of the practitioner to inquire into the changes which the curd of cheese undergoes in such instances, and to institute a comparison between its condition when altered by an excess of decomposing action and the more limited change in its particles. When affected by common decay, the researches of chemistry show us that in the decay of cheese certain volatile products arise from the comparatively fixed curd, and new bodies are formed and introduced into the system of whose mode of action we are supremely ignorant, unless when an overdose should happen to be followed by a fatal termination. Anatomy would lead us to infer that it is in some alteration in the natural constitution of the albuminous element of the food and blood, giving rise to an organic volatile produce, that we may probably look for the cause of those diseases which are characterized by a regular type, and constitute at present the *opprobrium medicorum.* It is not at all likely that what we term malaria—an expression analogous to instinct in relation to the actions of animals—is in its nature allied to mere gases, since the results which we can trace as produced by the action of such elastic fluids upon the animal system are irregular and reconcilable in general with simpler chemical action on the tissues or great systems of the organisms, and quite distinct from the regular phases of disease, as typified by such complaints as smallpox and measles. The circumstance that we have gained little or no ground for centuries in the investigation of the causes and consequent treatment of diseases which are distinguished by peculiar types, is a sufficient argument for reforming our methods of research, and for concluding that our scanty knowledge on such subjects may possibly have remained stationary in consequence of our having substituted in not a few instances words for ideas. In reference to the noxious influence of putrid game, and other decomposing forms of animal food, although little attention has been hitherto bestowed upon tracing evil consequences to such a source, it is scarcely necessary to say, that all will agree in the probable bad effects of such diet. The facts that over-salted food exclusively employed, is apt to induce scurvy, a state of the system in which the blood and tissues are imperfectly formed—that bacon in particular stages of decomposition and sausages have been known to produce death—that fresh dead bodies when excised communicate by inoculation dangerous disease to the human system—supply us with examples of injury, induced by an over-dose of noxious matter, and would lead us to the suspicion from analogy that a smaller dose cannot fail also to occasion a deteriorating action upon the organism, which is not the less influential because it is slow and silent in its operation. One of the great obstacles in the way of improvement in the medical profession, and particularly in the investigation of such diseases as we have just noticed, is the unfortunate love of argumentation instead of research which long ago crept in, and still adheres to the members of our noble science. We are quite willing to admit that the practice of meeting and discussing medical questions, is calculated to confer benefit on those engaged in it; but the propriety of that discussion being confined in general to raw and comparatively unlearned heads, and, above all, the wisdom of publishing such immature opinions, may safely be pronounced as highly questionable, irrespective of the injury inflicted on the youthful debaters. It has been well said that it is a very bad and dangerous habit to permit one's self to express an opinion regarding subjects which one has not *considered.* In this way, leanings are often contracted which continue through life, for nothing is more easy to acquire, or more difficult to cast off, than a mental bias. Many persons also can hardly engage in an argument without persuading themselves at least that that side is the better one which they defend. It is related that Pope Sextus V was impracticable to all but the Venetian ambassador. Those who investigated the art of the courteous politician discovered that when he wished to gain any point with the sovereign pontiff he began by proposing the opposite measure to that which he desired. This is too frequently the condition of our younger members, and having placed themselves in a false position, they often continue to occupy it through life.

It is too often the description of practitioners who oppose the results of what they denominate their experience to the efforts of exact science, to generalize or connect the isolated experiments or facts of the observer of disease. This conduct is very much on a parallel with that of the maker of any minute portion of the mechanism of a watch, who should refuse to allow it to enter as an element into the composition of the entire machine, by adducing trivial objections instead of giving his willing assistance to enable the work of his hands to occupy suitably the original position for which it was designed. It is a very common occurrence to find well and long-considered experiments objected to by crude and superficial arguments, which must have occurred also to the experimenter on his first survey of the subject, but which have long previously been overcome in his own mind. Often, too, they are assailed by those who are incapacitated from want of previous knowledge to give a correct judgment on the subject. We lately met with such an example in physical science, in the diseased leaven disseminated by a cheap press. The object of the writer, a Manchester Chartist, was to refute the first chapter of Genesis, for what purpose it is difficult to conceive, and the peculiar weak point for assault he considered to be the passages in which it is stated that light was created before the sun, just as if, added our wiseacre, "any one were ignorant that the sun is the source of light,"—thus exhibiting his want of acquaintance with the present theory of optical science, that the sun is not the source, but merely one cause of the development of the phenomena of light. It is this description of reasoners, who would have us to reject every good or sound idea, merely because it had been contaminated by a polluted source, as if gold were not all the purer from its oft-repeated subjection to the fiery ordeal.

It is in a conciliatory spirit, therefore, that we should address ourselves to such researches as have for their object the investigation of the very elements of our physiological knowledge. Such works as those we have cited at the commencement of this article are of this description. It may be that the conclusions contained in them are not always perfectly demonstrated; but if it be conceded that the authors have been labouring in the right direction, it cannot fail but that new light must be thrown on the subject of their investigations, by the number of facts which they have adduced and carefully scrutinised. It remains for the physiologist to exercise a critical eye upon such labours, not with a quibbling disposition, but with the judgment of one possessed of the spirit of generalization.

---

